# Female partners of patients after surgical prostate cancer treatment: interactions with physicians and support needs

**DOI:** 10.1186/1471-2296-11-19

**Published:** 2010-03-08

**Authors:** Jennifer M Evertsen, Alan S Wolkenstein

**Affiliations:** 1 University of Wisconsin School of Medicine and Public Health, Madison, Wisconsin, USA; 2 Center for Urban Population Health, Milwaukee, Wisconsin, USA; 3 Aurora University of Wisconsin Medical Education Group, Aurora Health Care, Inc, Milwaukee, Wisconsin, USA

## Abstract

**Background:**

Few studies have explored the women's experiences as a result of a partners' diagnosis of prostate cancer. This study begins to explore women's interactions with physicians (primary care and urologist) and the support needs associated with the diagnosis and treatment of their partners' prostate cancer.

**Methods:**

Two focus groups (n = 14) of women whose partners were diagnosed with prostate cancer (diagnoses' 1 - 18 months). A trained facilitator used open-ended questions to explore ideas. The framework approach was used to analyze the transcripts.

**Results:**

Three main themes emerged: 1. **More support**. Validation and information is needed for women including emotional support and opportunities to share experiences. 2. **Role of the physician**. The transfer of care once specialized treatment is no longer needed remained poorly defined, which increased confusion and feelings of abandonment related to the role of the primary physician. 3. **Partners' relationship changes**. Men became more dependent on their partners for support and to act as the primary communicator and caregiver.

**Conclusions:**

Additional research is needed in this field to confirm the importance of training primary care physicians to consider holistic treatment approaches that recognize the partner and family needs as important in the complete physical and emotional healing of their patients.

## Background

It has been well documented that a diagnosis of prostate cancer can affect the partner just as much as the individual diagnosed [1-4]. It is evident that female partners can experience high levels of stress and increased responsibility as a result of a diagnosis, yet there are often limited resources made available for family members to help cope with a diagnosis of this magnitude [3,4]. Good communication between the patient and their partner about the disease, its treatment, and emotional impact has been shown to be an important component to a more favorable adjustment to the diagnosis as it relates to the spousal relationship [3]. A small number of studies have assessed the quality of life (QOL) of both the patient and their partner and emphasize the emotional toll this diagnosis has on the couple [2,5]. In fact, one study noted that the QOL of both the patient and their partner was similar in relation to the psychosocial experiences associated with the diagnosis and treatment of prostate cancer [2]. Patients with a diagnosis of prostate cancer rely on a variety of resources for support during evaluation and treatment for couples, the impact of a prostate cancer diagnosis on the partner's personal health and well-being can be significant when there are limited resources available. Few published studies give a voice to the female partner affected by a diagnosis of prostate cancer as experienced at the level of the doctor-patient encounter. A systematic review examining the psychosocial adjustments of female partners to a diagnosis of prostate cancer found a small number of studies suggesting that partners report more distress than patients yet believe that patients are the more distressed [6]. Two studies, specific to breast cancer patients, noted that follow-up care by the primary care physician mainly assessed the patient's clinical needs and cancer reoccurrence but not psychosocial issues related to the patient or the partner [7,8]. So who is responsible for the **partner's **psychosocial issues during the diagnosis, treatment, and recovery of the patient's prostate cancer? A recent systematic review looked at the continuity of care in family practice and found that it is a "complex and multifaceted process" that is not always clearly defined [9]. The primary objective of this pilot study was to begin to explore the interaction of the female partner with the patient's physicians (primary care and urologist) and her support needs associated with treatment of and recovery from prostate cancer.

## Methods

Female partners of patients diagnosed with prostate cancer over the past two years and seen in a large Midwest urology clinic were eligible to participate in this study. Invitation letters by mail, informational flyers, and physician referral were used to gain permission of the patient. Patients were asked to pass along the information to their female partners if interested.

Focus groups using open-ended trigger questions were used to assess female partners' experiences with the urologist and primary care physicians during diagnosis, treatment and recovery. The Appendix lists some of these questions. Two focus groups were conducted on two different days, one in the late afternoon and one in the evening at the clinic where the male patients were receiving treatment. The male patients with the diagnosis of prostate cancer were not interviewed. A trained facilitator was present during both focus groups. The subjects signed an informed consent and were offered additional psychological support after the sessions if needed.

The focus groups were observed by an independent note taker, audiotaped, and transcribed. A seating diagram, in relation to the position of the facilitator was documented. Two independent researchers who were not present during the focus group discussion conducted the analysis. Data analysis was conducted using the framework approach of familiarization, identifying thematic framework, indexing, charting, and interpretation [10,11]. The annotating-the-scripts approach, involved reading the transcripts and writing interpretive thoughts about the data in the margins. The two researchers compared and reconciled the passages related to each theme; disagreements in themes were referred to a third researcher.

Wolkenstein's "Caring for Patients with Cancer" framework [12,13] that is a modification of the Kolb model [14] was used to further clarify emerging themes. This framework highlights the importance of reflective learning by the physician, patient, and family throughout the journey of caring for a patient with cancer including reflection at each stage of the patient encounter: 1) experiences, "A new world of new rules"; 2) losses and lamentation, "A new world of limited predictability"; and 3) transformation, "A new world."

Ethnography V5.08 software was used to identify and manage emerging themes [15]. Aurora Health Care Institutional Review Board approval was obtained for this research.

## Results

Fourteen female partners were interviewed in two focus groups (n = 14; group 1 = 7, group 2 = 7; non-Hispanic white = 12, Black = 1, unknown = 1, married = 13). The length of diagnosis of prostate cancer ranged from 1 to 18 months. The average age of participants was 61.6 years (range 47-77). All of the male partners had received surgical prostate cancer treatment prior to the focus group. Each group lasted 1.5 hours including the consenting process. None of the participants reported distress as part of the study process so no referrals to the psychologist were required.

Three distinct themes emerged from the focus groups including 1) support needs, 2) role of primary care and urologist and 3) changes to the couple's relationship.

### Theme 1: Support Issues and Needs

The participants identified two main areas of support needs: emotional and informational. Support came from a variety of sources including friends, primary care physicians, urologists, and support groups. Participants emphasized the need for additional support for themselves as well as their spouses. One woman said, "why isn't there a group for me to go to?" There was consistency in the fact that the women thought the male patients were reluctant to attend a support group and might benefit from mandatory or prescribed support groups to encourage greater buy-in and help validate some of their concerns. As one participant noted, "He won't ask for help, but he might accept it...there's been a great deal of denial."

The primary need identified by the participants was the need for more information. All of the participants believed that they did not receive enough information and were thrown into an unfamiliar role as a caregiver instead of a companion. This was highlighted by the fact that two woman mentioned that "The wife in something like this is very much involved," and "This diagnosis affects the wife as well." Some of the women said that "most of their questions were answered" but they also noted that they did not know what questions to ask which left them unprepared. They relied on the primary care physician and urologist to tell them what they needed to know, both the good and the bad. One woman said, "Maybe they don't tell us these {bad} things because they don't want us...to put ideas in our head." Another participant continued, "I think the men need to be told upfront...they need to come out and say...this can be a real problem. This is what's going to happen." Another said, "Is he {husband} the unusual one, or is he not the usual one?" One participant mentioned that she was interested in knowing as much as she could about the diagnosis and available resources while her husband didn't want to "face the reality." Some of the women suggested that they needed doctors, both the urologist and primary care physician, to be honest and give "more practical information" about how difficult and long the recovery is going to be. It was said "It's all trial and error...if you could share that information that would be helpful."

In addition to more information, the female partners identified a strong need for emotional support from family, friends, and others going through the same diagnosis. One participant said, ''When you find this out {diagnosis} you need to talk to somebody and that somebody's just not there.'' Discussion of shared experiences through support groups was mentioned by many of the women as important for both the patient and their partner to feel like what they were going through was normal. One of the women suggested the need for a public figure to serve as an advocate for prostate cancer and discuss the ''long road after treatment''.

### Theme 2: Role of Physicians (primary care and urologist)

Some of the participants said that the doctors ''were very respectful'' in regards to any questions that they would have but ''not really worried about me {emotionally} at all.'' A number of participants agreed with this statement and added:

" He {primary care doctor} would ask 'how are you doing' and you would say 'fine' and that's it. They don't ask anymore questions....I am not saying he doesn't take the time to talk to me, but he talks about the issue that I'm in there for."

Many of the women said that they tried to seek comfort from their own primary care providers but were disappointed with the lack of information and support they received. One woman said, "I have never talk with my family physician...I was never asked how I felt." Interestingly when asked whether the women received support from their husband's doctors, one women noted:

"...they {treating doctors}were not really worried about me at all. And I guess I wasn't surprised with that. I think actually... now that you've asked that question, I think I would have been very surprised if they had been worried about me. I just didn't...I mean, that never occurred to me. So that's an interesting question."

One woman noted that she was frustrated by the lack of ongoing support for her husband after surgery from his primary care physician and urologist. She said that she would bring her husband into the clinic with numerous symptoms and was told to wait and see what happens. A few participants, however, noted that they received more information and support from the primary care doctor then the urologist.

"He'll sit there with you...ask you about the family, everything. He'll say 'well, and how are you' and I have a bit of a habit of saying 'I'm OK' {Doctor says,} 'What do you mean by OK? Why don't you tell me what...why it isn't fine."'

".... He answered all the questions that we both asked, and we both had our list of questions and he would get them from both of us. You know, both of us would come at him with different types of questions, and we both felt very confident with the doctor."

"...we went in and we saw the primary care physician at that point and talked with him about the various options. So he was very helpful, very supportive. Obviously has some opinions but talked through all of the options, pros and cons, before we actually made the decision."

One woman noted that her partner was disappointed that his primary care physician did not visit him in the hospital after surgery. She noted, "His primary care just never came by to see him...he didn't stop by because he couldn't bill me."

General discussion emerged during the focus groups regarding inconsistencies in who should be providing follow-up, whether the urologist or primary care physician. One woman suggested that a team approach to the treatment and follow-up of prostate cancer could ease some of the burden on the patient and their family members. Several women mentioned

"...they were on their own until the next appointment."

"...once you're done seeing the urologist, you're still going back to your primary doctor. And if there are problems with one, the primary doctor needs to know about that. For further treatment down the line".

"If I have a question I would call the {urologist}. Those would be the first ones I would pick to phone and call". 

Two participants suggested the addition of a visiting nurse to help "relieve a little stress" by answering questions and assisting with care-giving duties.

### Theme 3: Relationship changes

During the course of the diagnosis and treatment many of the female partners said they felt as if they had taken on the role of the primary caregiver and were the sole emotional support for their partner. One woman said, "The wife in something like this is very much involved. It changes your life afterwards, not only the husband's, but yours." Another participant noted, "I can be there to listen, but I don't have any answers".

Many of the women emphasized that, at times, they had to communicate the emotional and physical struggles for their partner because he shut down. The lack of information and increased time serving as a caregiver rather than a couple caused increased stress for the female partner and at times took a significant toll on the couples relationship causing increasing tension and even arguing. One woman said that a high level of stress due to the diagnosis and treatment caused her to become physically ill with shingles and high blood pressure. She said,

"You just don't know where they're going to be from day to day. 'Cause one day they're one way, and the next day they're in a different mood and the next day...this is bothering them and that was where I wasn't prepared. I just...I knew we had to get through but I think you go through that big trauma period and then you hit a point where you go, 'okay, now we've got that sort of under control. We're not sure where we're going with it yet but it's kind of under control,' and that's when things sometimes get out of control. And you're their back at home, and they're going through all these different things emotionally. Well you're sucked into all that stuff. I mean, you don't know from day to day how they're going to handle it."

Another participant said, "We are still trying to figure it all out. It was a big change for both of us."

The couple's relationship during the diagnosis and treatment seemed to shift from the male patient attending doctor's appointments alone to the couples attending appointments together. One of the women lamented this relationship change when describing her partner's use of the words "we" and "us" to describe his illness. However, she noted a difference in classification when faced with her own illness. "When I had breast cancer, it wasn't 'our breast cancer. It was my breast cancer." A number of women expanded on this statement.

"From the very beginning of the process, we always did it together and I think it was just assumed... the two of us assumed that we would do it together."

"I didn't really get involved in going along on the visits until...you know, we got the point of the cancer diagnosis."

"I didn't have anything to do with the primary doctor and then when he had the diagnosis...after the biopsy then I went to the appointments with him."

Several women noted that physical changes during treatment and recovery caused changes to their relationship.

"{My husband} was never sick a day in their life...got a cold that would only last a day....there's been a great deal of denial."

"he had no symptoms...and you go to the doctor and all of a sudden, you're in the hospital and you come out feeling like hell, and you have incontinence and all of this, and ....you didn't feel like there was anything wrong with you".

"But everything's still not quite the same because now you're told you'll heal up, but when you have major surgery, your whole body goes through a change. And he seemed to like... he got moody and, you know, crabby, and so I just kind of went 'OK' cause you know... I don't know what to do".

"After two weeks my husband couldn't even get out of bed because of the pain...the discomfort...I mean I wasn't prepared for that".

One of the women said that her husband felt a "loss of manhood" due to a decrease of sexual function as well as the innate instinct to provide for his family during this time. Also, the physical effects of the diagnosis decreased the men's activity levels leaving the wife to take on more duties around the house.

"There's so much that's affecting their masculinity...."

"This is sixteen months later, we only go places where he can be near a restroom because he always feels like he has to go".

"I feel like a baby!...then you kinda feel like a women, you know, with a pad on".

"a women can't know what it's like mentally to a man not to be able to have sex and still have the desire".

" A man in his fifties...ending your sex life as you knew it, it's a big step. Especially with my husband, it was a big...a big blow to his ego and our sex life stopped as we knew it".

On another level, one of the women noted that her partner puts on his "social face" when around other people, including family, because he doesn't want them to know how sick he is.

## Discussion

It is evident that a diagnosis of prostate cancer can affect the female partner just as strongly as the patient. As noted in previous published research, women and their partners need additional emotional and informational support from their partner's urologist and primary care physician [1-4]. Our results are similar to those found by interviews conducted by Sinfield et al., which highlighted the perception of problems with care throughout the process of treatment and recovery including lack of specific information need's ensuring co-coordination between primary and secondary providers, and limited coping or support resources [16]. Our data highlights the emotional and physical effects that a diagnosis of prostate cancer can have on a spouse. It is increasingly important for treating physicians to recognize and reflect on these needs.

The participants identified specific needs and targeted several interventions in which their healthcare provider could play a more informative, integrated, and therapeutic role in the difficult process of renewal and healing. Interventions recommended included more informational resources at diagnosis and ongoing throughout treatment, referral resources for support groups and other individual advocates that could provide real-world reflections on their experience, clear definitions of transitions and who should be providing follow-up care, and a more patient-centered integrated care approach.

We found that the roles and transitions of the urologist and primary care provider are not clearly defined. Patients and their partners remain unsure about who is providing follow-up treatment and who is available to answer questions. More importantly, couples do not know enough about the diagnosis to be able to ask the right questions or know whether what they are experiencing is normal. In the meantime, it is important for both the specialist and primary care physicians to ensure that both partners are fully informed regarding the emotional and physical stressors faced during the diagnosis, treatment, and recovery process. Further studies need to be developed to understand the complex nature of the transition and role relationships among multiple providers.

Our data adds a unique look at the spouse's interaction with the primary care physician and urologist during the diagnosis, treatment, and recovery of prostate cancer. The participants highlighted the difficult journey from experiencing the initial diagnosis to the physical and emotional losses felt during and after treatment to the transformation of their lives to handle this new chapter. Many of the women said, after the formal focus groups had concluded, how appreciative they were to have had the opportunity to participate in this project because it gave them an opportunity to share and validate their feelings, ultimately allowing them to reflect on their past struggles. Wolkenstein et al. [12,13] defines this as "reflective learning," where an individual takes time to step back and reflect on the events leading up to a particular event and learn from the experience thereby transforming their self-concept. It is evident that there is no roadmap for women to follow as they face many question: "What is going to happen to us?" and "Who is going to be there when we need someone?" The women begin to question themselves and eventually their own "sense making" of the cancer experience for their partner, self, and their relationship.

This was a small qualitative pilot study and it included some limitations. First, it is difficult to generalize our results because of the small sample size and a single recruitment site. Secondly, additional data regarding time of diagnosis, stage of cancer, treatment and treatment outcomes were not specifically collected, as the purpose of this study was to summarize experiences with physicians throughout care and the range of stressors experienced by the partner. Thirdly, this sampling of women included only those whose partners had received surgical prostate cancer treatment, which could lead to very different experiences from other non-surgical options. Finally, we did not collect information on experiences from the providers' or husbands' perspectives, which could provide a complete, although more complex, picture of the relationship. Our research provides a quick snapshot of the opinions of a small group of women; further rigorous, longitudinal mixed-methods research needs to be conducted to clearly describe the complex relationship.

## Conclusions

This study provides additional reflection for urologists and primary care providers on the complex process of a diagnosis of prostate cancer and attempts to provide a greater understanding of the journey of experiences, losses/lamentation, and transformation that the female partner experiences as a result of her spouse being diagnosed with prostate cancer. There is a discovered need to facilitate new treatment options for couples' emotional recovery including counseling and support through primary care. The literature suggests that partners have difficulty with coping yet there appears to be little in the way of education and follow-up [17,18]. Additional research looking at different ethnic groups, different stages of disease and treatments, additional interviews with patients, family members, and physicians is needed to substantiate these results. It is important for urologists and primary care physicians to identify their roles in the diagnosis, treatment, and recovery phases of prostate cancer in order to lessen the burden of stress on the patient and their partner.

## Competing interests

The authors declare that they have no competing interests.

## Authors' contributions

JME was responsible for the data analysis, writing and preparing the manuscript for submission; ASW assisted with the study design, focus group implantation, data analysis and drafting of the manuscript. Both authors read and approved the final manuscript.

## Appendix

### Sample of Focus Group Trigger Questions

What role has your husband's primary physician played in this process? Also, has that role changed over time? Were you included in the visits to your husband's primary doctor? And how did that go?

How about YOUR primary care physician? Have you gone to your doctor since the diagnosis? What were your concerns? Did your doctor answer your needs?

Have you gone to the urologist with your husband? What was that like for you? Do you feel you were included in the decision-making and discussions of treatment options? Was your primary doctor open to discussing your feelings with him/her?

What are some of the things that you want from the doctors and are not getting? Can you describe what you need?

## Pre-publication history

The pre-publication history for this paper can be accessed here:

http://www.biomedcentral.com/1471-2296/11/19/prepub
